# Soft matter mechanics of baseball’s Rubbing Mud

**DOI:** 10.1073/pnas.2413514121

**Published:** 2024-11-04

**Authors:** Shravan Pradeep, Xiangyu Chen, Ali Seiphoori, David R. Vann, Paulo E. Arratia, Douglas J. Jerolmack

**Affiliations:** ^a^Department of Earth and Environmental Science, University of Pennsylvania, Philadelphia, PA 19104; ^b^Department of Mechanical Engineering and Applied Mechanics, University of Pennsylvania, Philadelphia, PA 19104; ^c^Norwegian Geotechnical Institute, Houston, TX 77043

**Keywords:** geomaterials, baseball mud, rheology, soft tribology, adhesion

## Abstract

Researchers looking for sustainable materials with optimal mechanical properties may draw inspiration from a baseball tradition. For nearly 100 y, a mysterious mud harvested from an undisclosed river site in New Jersey (USA) has been the agent of choice in the USA’s Major League Baseball for “de-glossing” new baseballs. It is unclear, however, what makes this “Rubbing Mud” work. Here, we perform a multiscale investigation of the rheology and tribology of this mud material under baseball-relevant conditions and identify three mechanisms by which the mud alters the surface properties of the baseball. First, the mud creates a more uniform baseball surface by filling in pores in the leather; this is possible because of its relatively high cohesion (clays and organics) making the material remarkably shear thinning. Second, the residue of cohesive particles coating the baseball effectively doubles contact adhesion. Third, a sparse population of angular sand grains are bonded to the baseball by clay-sized particles, leaving a studded surface that enhances friction. The proportions of cohesive, frictional, and viscous elements in Rubbing Mud conspire to create a soft material with an unusual mix of properties, that could find other applications in the development of sustainable geomaterials. Our improved understanding of the flow and friction of natural muds may also find use in modeling natural hazards such as mudslides and for locomotion in muddy environments.

## Significance

Since 1938, Rubbing Mud harvested from a secret river in New Jersey has been used to alter the gripping properties of baseballs in the USA’s Major League Baseball. In a game steeped in superstition, we sought to understand how and even if this mud actually works. We subject the Rubbing Mud to a series of mechanical tests that mimic its application, as well as its dynamics during gripping and throwing of a baseball. We found that the mud contains just the right mixture of clay and water to uniformly coat the baseballs with an adhesive residue, and just enough sand grains to enhance friction. The result is a material that spreads like skin cream, and grips like sandpaper. Understanding these remarkable properties may expand the use of sustainable natural materials for lubrication and gripping applications.

The complexity of soil is the key to its mechanical resilience; soil-based structures can withstand a wide range of stresses due to the multiscale interactions of cohesive agents (such as clay, natural stabilizers, geo- and biopolymers), frictional particles (e.g., sand), and moisture. There is a renewed interest in using locally sourced geomaterials to help meet the UN Sustainable Development Goals for resilient infrastructure and climate action, in view of cement’s carbon footprint and the global sand scarcity (1–3). Witness the expansion of rammed earth construction, which builds on ancient methods that take advantage of soil’s remarkable mechanical and thermal properties (4, 5). A lesser-known tradition is the use of soil-based materials as lubricants and friction agents. Since the 1950s, there has been one substance that every team in North America’s Major League Baseball (MLB) League has applied to baseballs for “de-glossing” and improving grip: Lena Blackburne Baseball Rubbing Mud, commonly called simply “Rubbing Mud” (6). This mud has a storied legacy. In 1938, the eponymous third-base coach of the Philadelphia Athletics discovered the Rubbing Mud in a Delaware River tributary near his home in New Jersey (USA). The precise location has remained a well-guarded secret since then (7). The proprietors have shared only limited information on how the mud is processed after harvesting: it is strained (mesh unknown), skimmed of excess water, rinsed with tap water, given an unspecified “proprietary treatment”, and allowed to settle (duration undisclosed) (8–10). Given the vagaries surrounding Rubbing Mud’s procurement and treatment, and the inconsistency with which it is applied to baseballs, MLB has made several attempts to replace it with “pre-tacked” baseballs. None of these substitutes have been satisfactory, however, leading MLB to issue strict instructions for proper application of Rubbing Mud (8). A natural question then arises: “What does the Rubbing Mud do?” We know of only one peer-reviewed scientific publication, which reported that Rubbing Mud had no significant effect on surface friction (11). In a game so steeped in superstition and tradition, it is unclear whether Rubbing Mud’s effects on the gripping properties of baseballs match pitchers’ beliefs.

It is useful to consider what is generally known about the physics of mud. Although the definition of mud itself is a bit squishy, it is usually considered as a dense suspension of predominantly clay and silt particles in water, with minor amounts of organic matter (12, 13). Sand is often present, but its concentration is small enough that it does not significantly influence flow behavior (12). There are two related but distinct phenomena that are relevant for understanding the Rubbing Mud problem: i) rheology, specifically its yielding and flow behavior under shear, which determines its ability to spread and coat the baseball; and ii) tribology, in particular, the friction and adhesion imparted on the baseball surface once mud is dried. The rheology of mud has been vigorously studied, because of its relevance for natural hazards, such as debris flows (14), and also for dredging and navigation purposes (15, 16). Natural muds, and model muds like clay suspensions (13, 17), are yield-stress materials that exhibit shear-thinning viscosity behavior. The yield stress arises due to interparticle attraction, which forms cohesive aggregates; thus, yield stress increases with both the volume fraction of clay particles and the strength of interparticle bonds (18). When sufficiently sheared, aggregates break up and align with the flow resulting in shear thinning; the viscosity decreases with increasing shear rate. In terms of tribology, there are many studies regarding friction experienced by objects on and in granular substrates due to their importance for biological and robotic locomotion (19); however, these studies have mostly focused on cohesionless materials (sand) (20). Motivated by the problem of dust deposition on solar panels in humid environments, researchers have demonstrated that dried mud increases adhesion and friction of glass surfaces (21, 22). In experiments examining evaporation of polydisperse suspensions, our previous work showed how small particles condense within shrinking capillary bridges—ultimately creating solid bridges of clay-sized particles that bond larger (silt- and sand-sized) particles to surfaces (23).

**Fig. 1.**
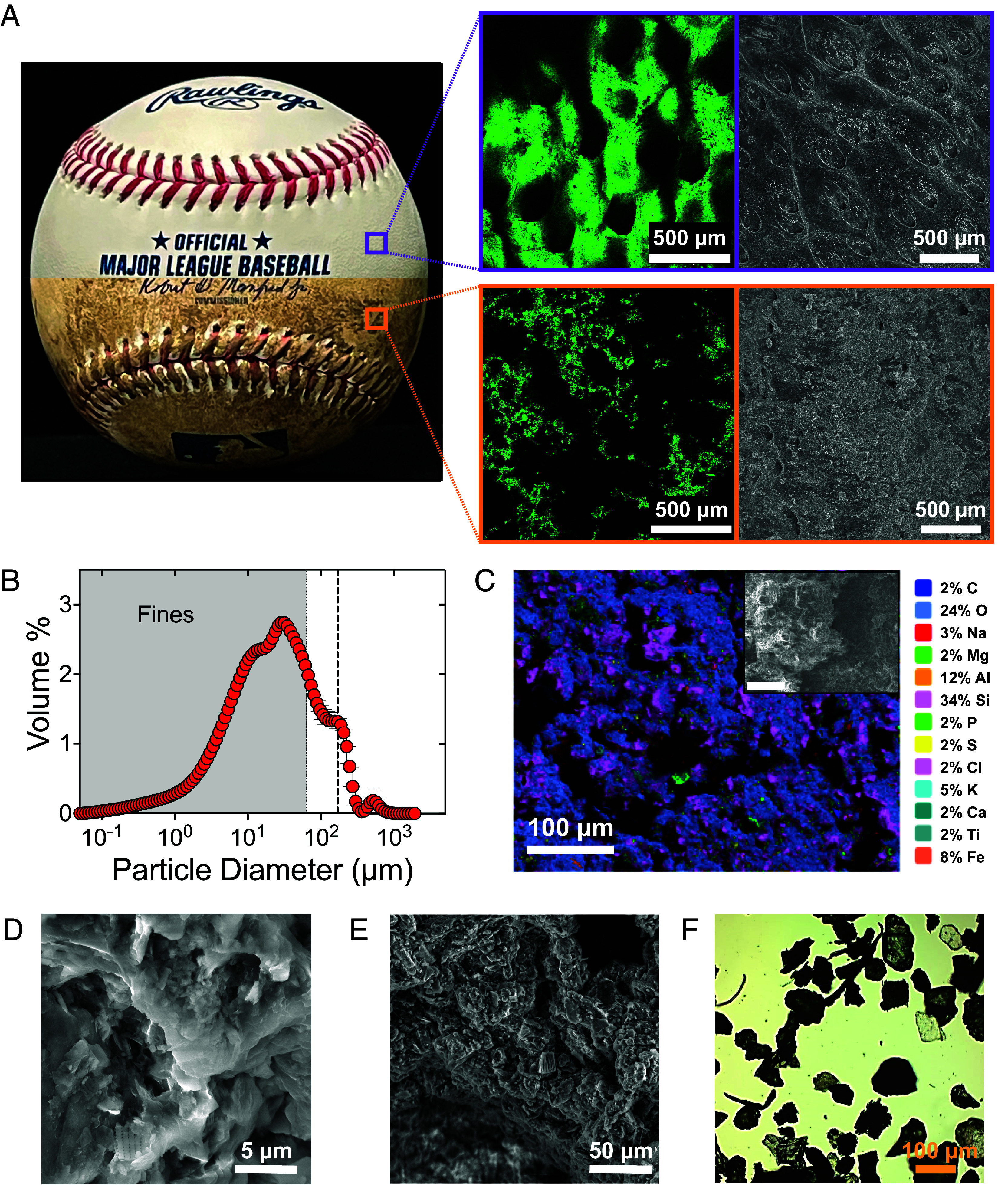
Rubbing Mud composition and microstructure. (*A*) The microstructural visualization of a clean baseball surface (*Top*) and a mudded baseball (*Bottom*), using confocal laser scanning microscopy (CLSM) (green images), and scanning electron microscopy (SEM) (gray images). (*B*) Particle size distribution of Rubbing Mud. The dashed line indicates the suspected mesh size used for the processing of Rubbing Mud; the gray box indicates the range of particles considered “fines” (≤62 μm). (*C*) Energy dispersive X-ray spectroscopy (EDS) shows a qualitative map of the elements present in Rubbing Mud smeared on a baseball; the *Inset* shows SEM image of the same area as the EDS map. SEM images of clay sheets and silt particles that contribute to the (*D*) tail and (*E*) the peak of the particle size distribution. (*F*) Optical microscopy of angular sand grains and silt-size particles with plant detritus that correspond to the coarse fraction.

The above studies lead to the following inferences about Rubbing Mud: it has shear thinning behavior that allows it to spread into a thin and uniform coating; its clay and organic content enhances adhesion once applied; and any sand-sized particles may be bonded to the baseball surface by clay-sized particles as the mud dries, leading to increased friction. To test these ideas, here we perform a multiscale experimental approach on Rubbing Mud to characterize its rheology and tribology under conditions that are relevant for baseball application and play. Shear rheometry tests show that Rubbing Mud is a nearly jammed yield-stress material with remarkable shear thinning behavior, akin to a skin cream or polymer melt. Scanning Electron Microscopy (SEM) reveals that the mud coats the baseball uniformly (Fig. 1*A*), smoothing the surface by filling in holes on the pristine baseball surface. Atomic Force Microscope (AFM) tests demonstrate that dried mud enhances adhesion. A unique soft tribology experimental setup, that mimics human fingers, coupled with microstructural visualization using Confocal Laser Scanning Microscopy (CLSM) show how angular sand grains bonded to the surface increase friction—up to the point that sand grains are sheared off at high sliding speeds. Our observations unravel most of the mystery behind the constituents of Rubbing Mud and its (micro)mechanical functions.

## Results

### Rubbing Mud Composition and Microstructure.

The material we examine here is from a single container of Lena Blackburne Baseball Rubbing Mud (Personal Size, New Jersey, USA) purchased in January 2022, except for X-ray Diffraction (XRD) analysis that was conducted in the year 2012 on a container purchased then (see below). The material has a dark brown appearance and has the feel of skin cream; a dollop placed in the hand is stable, but it smears easily onto surfaces and does not feel gritty. The weight percent of solids is approximately 57%, the balance being water. If we assume a typical sediment density for silicates (2.65 g/mL) and that particles are nonswelling—both assumptions that are likely violated at least somewhat for this heterogeneous material—this corresponds to a solids volume fraction of approximately 33% (*ϕ* ≈ 0.33). We measured the particle size distribution of the mud using a laser diffraction device (*Materials and Methods*); mud samples were suspended in aqueous solution and subjected to sonication to break up aggregates. We also sieved the mud at a mesh size of 75 μm, in order to image the smaller (with SEM) and larger (visual images) particles to determine their composition. The particle size distribution is polydisperse, ranging from nanometer to millimeter scales (Fig. 1*B*). Some notable features of the distribution are a relatively heavy tail of very fine-grained materials (<10 μm) that likely represent individual clay particles; a broad peak (perhaps two merged peaks) in the 10 μm range that appears to be clumped stacks of clay particles and individual silt grains (Fig. 1 *D* and *E*); a shoulder in the fine sand range (∼10^2^ μm) that corresponds to angular sand grains (Fig. 1*F*); and finally the smallest peak close to ∼10^3^ μm which seems to be the hydrodynamic size associated with the sparse, rod-like plant detritus (Fig. 1*F*). In many ways this distribution is similar to other natural mud (14). There is one conspicuous feature, however; the sharp dropoff in occurrence of grains larger than roughly 169 μm (Fig. 1*B*). We suspect that this dropoff corresponds to the sieve size used for processing of Rubbing Mud and that the residual peak corresponding to larger plant detritus survives because those elongated particles have a minor axis diameter that is capable of passing through the mesh.

We performed CLSM and SEM imaging of the microstructure and texture of a newly purchased “clean” MLB-licensed baseball (Rawlings), and a “mudded” baseball following an application protocol similar to what is recommended by MLB (*Materials and Methods*). The clean baseball is riddled with ellipsoid-shaped surface pores, with average length ∼300μm, width ∼150 μm, and depth ∼55 μm, but its surface is relatively smooth at smaller length scales (Fig. 1*A*). The mudded baseball is the opposite; there is relatively enhanced roughness at small scales, but the mud appears to fill in pores and create a more uniform texture at larger scales (Fig. 1*A*). Rubbing Mud leaves a thin layer of particles with a range of sizes stuck to the baseball surface; surface texture indicates that most of these particles are clay aggregates or silt (Fig. 1*C*).

We performed Energy Dispersive X-Ray Spectroscopy (EDS) to obtain qualitative information on elemental composition of the mudded baseball surface (Fig. 1*C*). The most dominant elements are silicon (Si) and oxygen (O), which indicates that the vast proportion of particles are silicates (quartz and clay minerals). Silts and sands of the southern New Jersey coastal plain are known to be almost entirely quartz (24). The presence of aluminum (Al), iron (Fe), and potassium (K), and trace amounts of magnesium (Mg), are consistent with previous analysis of river sediments in the area (25) and the clay minerals (e.g., kaolinite, smectite, illite) reported in regional soils (24). The small carbon (C) content observed in EDS is consistent with the observed 5% “loss on ignition” weight loss experienced by the Rubbing Mud under combustion, indicating a minor amount of organic material. Some of this organic material is the observed plant detritus, but an unknown amount is almost certainly associated with biopolymers that we do not characterize here. We performed XRD analysis to supplement EDS. Results indicate that roughly half of the mud by weight is quartz and that mica makes up a small fraction of crystalline species. The balance of the sample appears to be dominated by clays. Although XRD peaks are not strong enough to determine clay mineralogy (*SI Appendix*, Fig. S6), observations are consistent with mixed-layer clays that are typical of river sediment: smectites mixed with mica, kaolinite, and illite (26–29). The EDS and XRD results are thus internally consistent. Combined with the grain size data, we infer that the mud is roughly half clay with the other half composed of predominantly quartz silt and sand.

Soils and sediments are often categorized by their percent fines, corresponding to sizes ≤62 μm, because these materials exhibit cohesion (30). This cohesion typically arises due to electrostatic effects, whose relative strength (compared to particle mass) increases with decreasing grain size (30, 31). The vast proportion of particles present in Rubbing Mud are fine particulates. A common way to characterize particles’ electrical charge in water is zeta potential. We measured a zeta potential of approximately −25 mV for Rubbing Mud, which indicates a weakly attractive suspension susceptible to aggregation (32). Some additional cohesion is likely arising from biopolymers that are ubiquitous in natural mud (33), as well as the unknown “proprietary treatment” added in processing. In geotechnical terms, Rubbing Mud could be classified as a highly plastic silt-based soil (34, 35). From a soft matter perspective, the ability of Rubbing Mud to withstand its own weight (without flowing) at relatively low volume fraction (*ϕ* ≈ 0.33) places it in the category of a “soft solid,” like a gel. For comparison, suspensions with hard-sphere like particles exhibit solid-like behavior only when volume fraction approaches *ϕ* > 0.55 (36, 37).

Once dried, however, the Rubbing Mud leaves a residue of predominantly cohesive fines, with sparse sand grains that stud the surface of the baseball. The effect of this residue is that the surface of the baseball feels more “gritty.” It is common lore that one way Rubbing Mud de-glosses a baseball is by scuffing the surface (7). These observations motivate a more systematic study of flow and friction behavior of Rubbing Mud.

### Macroscale Flow Tests.

**Fig. 2.**
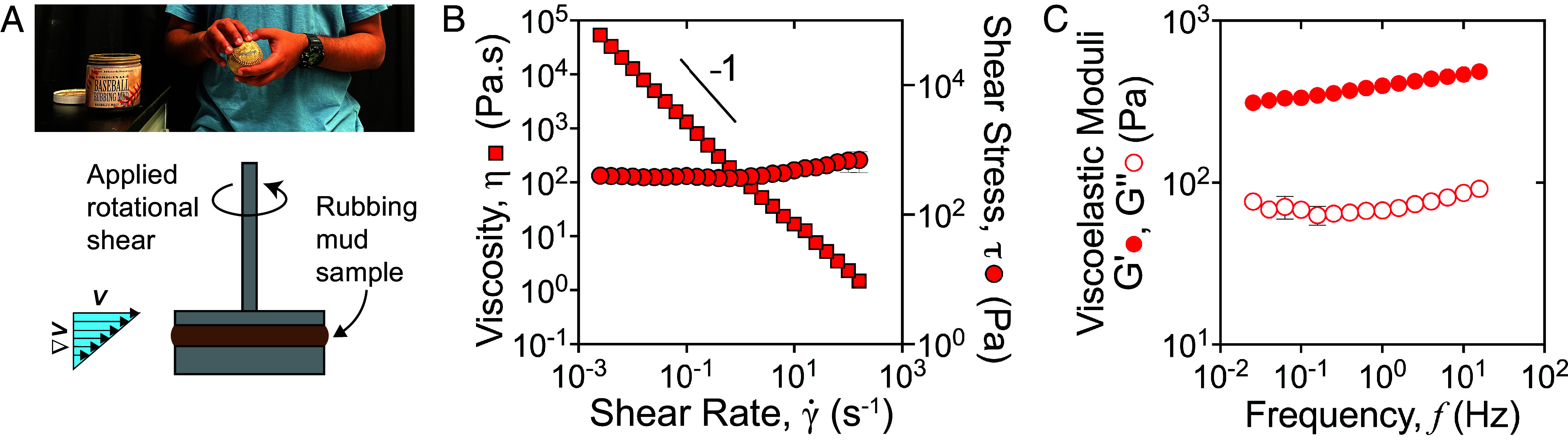
Rheological characterization of Rubbing Mud. (*A*) Picture of the process of rubbing Rubbing Mud onto the baseball surface (*Top*) and a schematic of the parallel-plate experimental setup (and corresponding flow directions) used to measure flow properties of Rubbing Mud. The spin rate of the top plate determines the velocity *V*, and the shear rate $\dot{\gamma }\equiv \nabla V\approx V/h$, where *h* is the gap thickness. (*B*) The steady-shear flow curve, where the viscosity and shear stress are plotted as a function of applied shear rate, for Rubbing Mud. The slope ≈−1, implies that $\eta ∼{\dot{\gamma }}^{n-1}$, where *n* ≈ 0 at low to medium shear rate values. (*C*) Linear viscoelastic properties, elastic ($G^{\prime }$) and viscous ($G^{\prime \prime }$) moduli, as a function of applied oscillatory frequency (*f*). Data show that Rubbing Mud is a *soft material* with viscoelastic behavior.

Rubbing Mud flow properties were characterized using an advanced strain and stress control rheometer (TA Instruments, DHR-3) with plate–plate geometry (Fig. 2*A*). All experiments were carefully performed to minimize material slip and confinement effects (*Materials and Methods*). We have previously used this configuration to examine the rheology of natural mudslide materials (14), and also model earth materials composed of mixtures of kaolinite clay, sand, and water (38). One way to assess material flow behavior is a steady shear protocol, in which the sample is subjected to a fixed (shear) strain rate ($\dot{\gamma }$) and a steady-state shear stress (*τ*) is measured; an effective shear viscosity is determined as $\eta_{\mathrm{eff}}\equiv \tau /\dot{\gamma }$. The experiment then sweeps through a range of strain rates to produce a “flow curve” of viscosity (or shear stress) as a function of $\dot{\gamma }$. Unlike Newtonian fluids (e.g., water, oils) that possess constant viscosity, Rubbing Mud displays strong shear-thinning viscosity behavior; the material viscosity follows a power-law relationship $\eta_{\mathrm{eff}}\propto {\dot{\gamma }}^{n-1}$, where *n* is a power-law index. Results show that *n* ≈ 0 for most of the sweep, with deviations at high values of $\dot{\gamma }$ (Fig. 2*B*, *Left* axis). In other words, viscosity scales inversely with shear rate, decreasing from a value roughly 100 times larger than peanut butter at the lowest shear rates, to a value comparable to cooking oils at the high shear rates.

The flow data also indicate that Rubbing Mud exhibits marked yield-stress behavior. Yield stress can be considered as the shear stress that must be overcome to cause a material to flow and is determined as the residual stress in the limit of vanishing shear rate. Here, the mud shear stress remains independent of shear-rate (*n* ≈ 0) and relatively constant, $\tau_{y}\approx 10^{2}$ Pa, for a wide range of $\dot{\gamma }$ (Fig. 2*B*, *Right* axis), where *τ*_*y*_ is the Rubbing Mud yield-stress value. This value of *τ*_*y*_ is comparable to skin cream (39), and also similar to nearly jammed mudslide materials (14) and dense kaolinite-clay suspensions (38). Overall, Rubbing Mud flow behavior (i.e., shear-thinning, yield stress) is reminiscent of polymer melts and gels (40), which often display both fluid-like and solid-like behavior depending on how fast they are sheared (41). The flow curve is very similar to our previous observations of an idealized model mud—a nearly jammed suspension of kaolinite in water (*ϕ* = 0.40) (38) (*SI Appendix*, Fig. S2)—and also to that reported for skin cream (39, 42). Note that both facial mud treatments and skin creams are designed to shear thin in order to enhance spreadability (39, 42). To understand how the relative proportions of water and solids influence rheology, we performed additional steady-shear experiments with serially diluted Rubbing Mud. These tests show a rapid drop in yield stress and viscosity as water is added, and a corresponding rapid decline in the rate of shear thinning for diluted mud (*SI Appendix*, Fig. S3). This drop indicates that the Rubbing Mud is likely processed by the proprietor to be near the jamming point; i.e., its water content is intentionally tuned so that the mud is a marginally stable solid with maximum shear thinning.

We also performed oscillatory rheometry at slow (quasi-static) rates, over a range of frequencies *f*, to determine the storage (solid-like) and loss (fluid-like) modulus of Rubbing Mud (*Materials and Methods*). The principle is to probe deformation in the solid-like state of the material, and determine how much of this deformation is accommodated by elastic strain (storage modulus, $G^{\prime }$) and viscous dissipation (loss modulus, $G^{\prime \prime }$). The zeroth-order observation is that $G^{\prime }\gg G^{\prime \prime }$ which indicates that material deformation is predominantly elastic when slowly deformed, while the value $G^{\prime }∼O\left(10^{2}\right)$ Pa confirms that Rubbing Mud is a soft solid (43). The first-order observation is that storage and loss moduli are nearly constant across the frequency *f* range investigated here and, on average, $G^{\prime }$ remains two to three times greater than $G^{\prime \prime }$. This rheological behavior is consistent with a linear viscoelastic material (Fig. 2*C*) at relatively low strains, as reported in gels formed from weakly interacting particles (44). The trends and magnitudes for storage and loss modulus are very similar to observations of natural fine-grained mud (15) and skin cream (39). Taken together, our rheologic investigation of Rubbing Mud reveals that it exhibits flow properties that rival purpose-designed cosmetic products in terms of spreadability, allowing the mud to evenly coat the baseball in a thin layer.

### Mesoscale Soft Tribology.

**Fig. 3.**
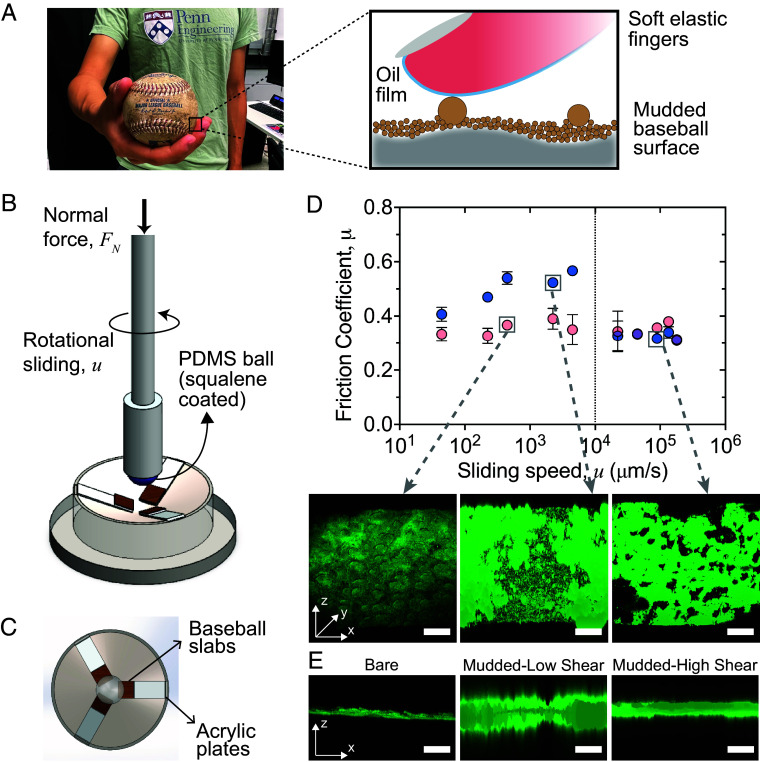
Soft tribological characterization of Rubbing Mud friction on baseball leather. (*A*) Photo of a human hand gripping the baseball surface (*Left*) and the constituent interfaces at play (*Right*). (*B*) The rheo-tribometer customized to mimic the hand gripping a baseball surface. Experiments are conducted at a constant applied normal load, and the rotational speed of the PDMS sphere at the top is varied to generate different sliding speeds. The ratio of the sliding shear force and the applied normal force is then calculated as the frictional dissipation of the system. (*C*) The top view of the setup with an illustration of the cut-out slabs of baseball leather that are glued to the bottom acrylic plates, which are then attached to the bottom geometry. (*D*) The change in friction coefficient (*μ*) as a function of applied sliding speeds (*u*), for a squalene-dipped PDMS sphere in contact with clean (pink) and mudded (blue) baseball leather under an imposed $F_{N}=10$ N. The clean baseball has a constant low *μ* at all sliding speeds, while the mudded baseball shows enhanced *μ* that increases with *u* up to $u\approx 10^{4}\;\mu$m/s. Beyond that speed, the friction drops to the value of a clean ball; the confocal images (at an angle of ∼20^°^ from the x–y plane of reconstructed 3D image) show the change in microstructure between a clean and mudded baseball, and the removal of larger particles from the “scrubbed” baseball surface at high-speed shear. (*E*) The vertical *z* profile of the reconstructed 3D images of samples (left to right) that show the changes in lengthscales from bare to mudded to scrubbed. (Scale bar, 500 μm.).

To measure the surface friction on clean and mudded baseballs, we designed a custom ball-on-plate soft tribology apparatus that was attached to the shear rheometer setup (Fig. 3*B*). This setup is used to impose a constant normal force *F*_*N*_ on the sample, and then shear at prescribed sliding speeds (*u*) and measure the shear force *F*_*s*_. The effective friction can then be estimated as $\mu \equiv F_{s}/F_{N}$. Assessing the effect of Rubbing Mud on the surface friction of a baseball requires special attention. Pieces of the baseball leather are mounted to the acrylic base plates, and a ball is lowered to contact the surface (Fig. 3*C* and *SI Appendix*, Fig. S1). Our experiments started with a standard steel ball. The steel ball-on-baseball tribological characterization shows that the friction coefficient is independent of the applied normal load, when the baseball surface is mudded. We speculate that the Rubbing Mud created a more uniform surface on what was otherwise a rough surface with valley-like structures (*SI Appendix*, Fig. S4*A*). However, a steel ball does not mimic the elastic properties of skin, so we replaced it with one made of PDMS. The elastic modulus of the PDMS was tuned to match that of human fingers (≈1 MPa) (45, 46). We found that the PDMS was abraded by the dried mudded baseball surface under shear (*SI Appendix*, Fig. S4*B*). Therefore, for all experiments reported here, the PDMS ball was coated with synthetic squalene. This has a twofold advantage: i) squalene is a major component of sebum secreted by human oil glands (47, 48), thus mimicking finger lubrication; and ii) the coating minimizes the surface wear of the PDMS spheres. Squalene-coated PDMS spheres are brought into contact with the baseball surface to characterize surface friction properties imparted by Rubbing Mud. This combination of PDMS ball, squalene, and mudded baseball at various sliding speeds is designed to mimic the situation of a pitcher gripping and throwing a baseball (Fig. 3*A*).

Here, we report friction measurements under steady shear, for a representative experiment at a constant normal force $F_{N}=10$N that is the same order of magnitude as a pitcher gripping a baseball (49), and a safe normal load where multiple sliding speeds can be tested in our tribo-rheometer setup. For a clean baseball, the friction coefficient maintains μ=0.33±0.05 over a four-order-of-magnitude increase in sliding speed (Fig. 3*D*). This indicates that friction would be the same whether one is statically gripping the baseball, or throwing and spinning the baseball. Results are very different for a mudded baseball. Friction is enhanced at most sliding rates, relative to a clean baseball control (Fig. 3*D*). In fact, as sliding speed increases so does friction, reaching a peak value *μ* ≈ 0.6 at a sliding speed of ∼10 mm/s. Beyond that speed we observe an abrupt drop in friction for the mudded baseball, so that at the highest sliding speeds *μ* is comparable to a clean baseball. This implies that when sheared sufficiently hard, the condition of the surface for a mudded baseball becomes similar to a clean baseball—i.e., it behaves as if the mud is no longer there. Indeed, CLSM images taken before and after the tribology tests show that the mud is detached from the baseball by high-speed shearing (Fig. 3 *D* and *E*). Tests with a lower imposed normal force, $F_{N}=1$ N, were also performed (*SI Appendix*, Fig. S5). The friction coefficient *μ* increased on average compared to the bare baseball but did not vary across the sliding speeds *u*.

In summary, Rubbing Mud increases the friction of the baseball surface under conditions relevant for pitching. Results indicate that this friction boost is enhanced for throwing/spinning compared to statically gripping a baseball—up to a factor of two larger than a clean baseball. Sufficiently large sliding speeds and high normal load appear to knock bonded particles off of the mudded baseball, effectively scrubbing the surface and leaving the baseball in a condition comparable to a clean one.

### Nanoscale Material Adhesion.

**Fig. 4.**
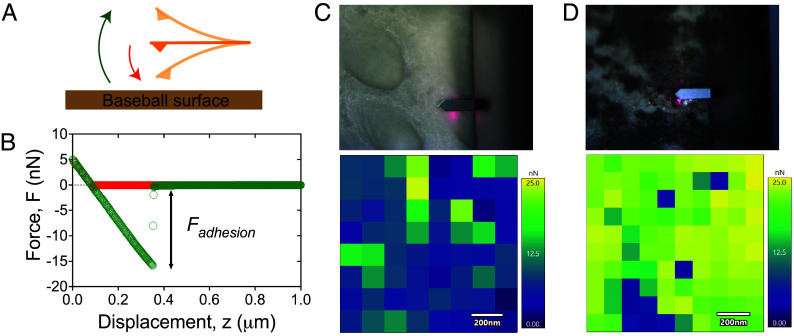
Adhesive properties of Rubbing Mud at the nanoscale. (*A*) Illustration of the approach (in red) and retraction (in green) of the AFM tip on the baseball surface. (*B*) Representative force curve from an AFM experiment [same color scheme as (*A*)]. The adhesive force is measured as the peak retraction force. Optical microscope images of the probing sample with the AFM cantilever (*Top*), and representative force maps (*Bottom*) on a sample size of 1 μm × 1 μm, on (*C*) a clean baseball surface and (*D*) a mudded baseball surface. On average, the adhesive force on a clean baseball is half that of a mudded baseball. The optical images show the contrast between the surface pores on a clean baseball surface and the sand particles (shiny substance) dispersed across the clay matrix (dark substance) on a mudded baseball surface. In the latter case, AFM measurements are performed only on the dark substance, which primarily contains clay and silt particles.

Besides friction, adhesion is the other relevant property to understand in terms of material grip (50). We measured adhesive forces on clean and mudded baseball leather using atomic force microscopy (AFM; *Materials and Methods*). Given the constraints of the technique, these experiments could not be conducted with squalene. Thus, while we are uncertain how our measurements might translate to conditions of game play, they at least allow a relative comparison that helps to understand how mud may alter the stickiness of baseballs. We employed a standard soft cantilever tip: silicon nitride material, with a spring constant *k* = 0.055 N/m. The adhesive force is estimated as the difference in force between the approach and retraction of the cantilever tip (Fig. 4 *A* and *B*). This measurement is taken at 100 different locations within a 1 μm × 1 μm area to generate a force map (Fig. 4 *C* and *D*). It is worth noting how small the observation area is; it is smaller than the typical size of a single particle in the mud. For the mudded surface, the AFM tip was intentionally placed to avoid sand particles on the surface. We may expect some spatial heterogeneity in adhesion across mudded leather, and indeed over clean leather as well.

The force map on the clean baseball leather shows some variability in adhesion, with an average value of 9.6 nN (Fig. 4*C*). The mudded baseball surface has consistently higher adhesive force, with an average (≈20 nN) that is double the value of the clean surface (Fig. 4*D*). Given that the AFM test location was chosen to avoid large grains, the enhanced adhesive force for the mudded surface is associated with fine cohesive particles attached to the surface.

## Discussion and Implications

The particulates in Rubbing Mud are predominantly silt and clay, with minor amounts of sand and organic material. The clay, fine silt, and organic matter make the mud cohesive (sticky), while the sand—which is relatively large and also angular—acts as a grit. From a soft matter perspective, Rubbing Mud is a nearly jammed cohesive suspension that has the properties of a skin cream; this allows it to be held in the hand like a solid, but also spread easily to penetrate pores and make a very thin coating on the baseball surface. Coarse particles are sufficiently sparse that they do not appear to influence the flow behavior of Rubbing Mud. When the mud dries on the baseball, however, the residue left behind is quite different from skin cream. Angular sand particles, bonded to the surface of the baseball by clay, increase surface friction by up to a factor of two. Our earlier work suggests that this bonding arises from solid bridging that occurs during drying (23). On the other hand, cohesive fine particulates effectively double the adhesion. The relative proportions of cohesive particulates, frictional sand, and water conspire to make a material that flows like skin cream, but grips like sandpaper. One additional effect of the mud is that it may make the baseball “feel” more uniform, and reduce variation in surface texture from one baseball to another (*SI Appendix*, Fig. S4). The mudded baseball is rougher at smaller scales due to the particles but is actually smoother at larger scales; the mud fills in holes and other imperfections in the factory baseball (Fig. 1*A*). While manufacturing practices and uniformity of baseballs have varied significantly over the years (51, 52), the Rubbing Mud has been a consistent surface treatment.

Our findings on how Rubbing Mud improves baseball grip lead to a question: is the Rubbing Mud special, or would mud from other places perform similarly? The size and chemical composition of particulates in Rubbing Mud is not unusual for muddy tidal environments (30), with one exception: the sharp cutoff in occurrence of grains >169 μm makes the particle size distribution more asymmetric than what is typically reported for natural mud (30). As mentioned, we suspect that this is the result of sieving during processing; the raw river mud likely contains a larger fraction of sand. The yield stress of Rubbing Mud is similar to other natural muds previously reported (14, 15, 18, 30), which vary widely in particle composition and water content. Because yield stress depends on both the concentration and interparticle potential of fines, it is instructive to compare our results to simplified model mud mixtures. Pure kaolinite suspensions achieve a yield stress similar to Rubbing Mud’s, ∼$O(10^{2})$ Pa, at a comparable volume fraction (*ϕ* ≈ 0.3) (18, 38). The measured electrical charge of Rubbing Mud suspended in water is also similar to kaolinite suspensions (23). Although our estimate of *ϕ* ≈ 0.33 for Rubbing Mud is crude, this comparison suggests that the interparticle attraction and resultant cohesion in Rubbing Mud is comparable to a simple clay suspension. In other words, Rubbing Mud is not special in this regard. It is also not particularly special in terms of its shear thinning behavior. Virtually all muds and model-mud materials studied previously exhibit shear thinning (14, 15, 18, 30, 38), a phenomenon also observed in gels, creams, emulsions, and many colloidal suspensions (41). While the inverse relation between viscosity and shear rate over six orders of magnitude is impressive, we observe the same behavior in the model mud system of a nearly jammed kaolinite–water suspension (38) (*SI Appendix*, Fig. S2). While natural biopolymers, and any unknown additives mixed in during processing, likely have some impact on the quantitative values for yield stress and viscosity, the qualitative rheologic behavior of Rubbing Mud is entirely consistent with a clay-rich suspension in the vicinity of the jammed state. Returning to our question of whether Rubbing Mud is special, the answer is no and yes. If there is something special about Rubbing Mud, it is in the proportion of the basic ingredients—cohesive particles, frictional particles, and water—rather than the ingredients themselves. The rheology of soft Earth materials is exquisitely sensitive to the proportions of these ingredients (38, 53, 54). We speculate that similar tidal-creek environments with similar fine-grained sediment deposits would also make adequate raw material and that there are two important steps in processing this to make Rubbing Mud: i) sieving the mud to remove enough coarse material so that its rheology is dictated primarily by cohesion (and not frictional interactions) (18), while leaving enough sand of the appropriate size so that solid bridging by clays during drying (23) produces a baseball surface studded with sand grains; and ii) adjusting the volume fraction of the mud so that it is nearly jammed. We guess that the “proprietary treatment” (8) added to Rubbing Mud involves substances meant to stabilize, preserve, and/or disinfect the mud.

The components of Rubbing Mud are mundane, but its mechanical behavior is remarkable in ways that could find numerous applications in the quest to replace synthetics with sustainable geomaterials. First, its monotonic and predictable shear-thinning means that this mud could be a very effective lubricant, if gritty sand is removed. Mud could also be used as a friction agent for improving traction on slippery surfaces in remote field settings, perhaps by optimizing the fraction of sand without diminishing spreadability. The combination of enhanced friction and adhesion of dried mud also suggests that it could find use as a binding agent, to improve the properties of locally sourced geomaterials in situ for construction. The knowledge gained here regarding friction and adhesion properties of mud could also find use in understanding the interactions of feet, wheels, and animals with wet and dry muddy substrates (20, 55–57). As for the future of Rubbing Mud in Major League Baseball (8), unraveling the mystery of its behavior does not and should not necessarily lead to a synthetic replacement. We rather believe the opposite; Rubbing Mud is a nature-based material that is replenished by the tides, and only small quantities are needed for great affect. In a world that is turning toward green solutions, this seemingly antiquated baseball tradition provides a glimpse of a future of Earth-inspired materials science.

## Materials and Methods

### Baseball and Mud Material.

The baseball rubbing mud was purchased from Lena Blackburne Baseball Rubbing Mud, Maple Shade, NJ. The material was used in our experiments as received, without further modifications. The baseballs used in the studies were the “Official Major League Baseball” model purchased from Rawlings Sporting Goods. For the tribological and adhesion experiments performed, the Rubbing Mud was rubbed on the peeled baseball skin surface using hands and left overnight to dry in ambient conditions. This protocol mimics the time scale in regular baseball games.

### Particle Size and Elemental Analysis.

Particle size distribution was characterized using a Beckman-Coulter Particle Size Analyzer LS13-320. Grain size was determined in 114 log-spaced bins over the range 0.04 μm to 2,000 μm. Microscopic images of the bare and mudded baseball surface were obtained using a scanning electron microscope (FEI Quanta 600 Environmental Scanning Electron Microscope). The images were acquired at an accelerating voltage of 15 kV and a water vapor pressure of 0.75 torr. The setup is also equipped with an energy dispersive EDS that allowed for chemical characterization via element mapping and semiquantitative compositional analysis.

### Imaging 3D Microstructure.

A high-speed confocal laser scanning platform (Leica Stellaris 5 model) equipped with a modular DMi8 inverted microscope was used as the optical tool to visualize the 3D microstructure of the baseball surface. Drops (≈1 mL) of the dye solution, containing 1 wt% of Rhodamine B dye ($\lambda_{\mathrm{ex}}=546$ nm and $\lambda_{\mathrm{em}}=567$ nm) in deionized water, were applied on top of the baseball surface and dried overnight for the dye particles to be adsorbed onto the bare/mudded baseball surface. Confocal microscopy was then performed using dry objectives (5×) to visualize the microstructure. Images of size 3,000 μm × 3,000 μm were obtained at vertical distances Δz = 30 μm to capture the three-dimensional microstructure. The final 3D images were reconstructed from the obtained 2D image stacks using the open-source image analysis platform Fiji (58).

### Rheological Characterization.

Rheological characterizations were performed in an advanced strain and stress control rheometer (TA Instruments, DHR-3), using a 40 mm parallel-plate setup at 25^°^C. To reduce the sample slip effects at the boundaries during our measurements, both the top and bottom plates were modified by attaching 50-Grit size serrated surfaces. This is equivalent to roughness length scale ≈300 μm, which is the same order as the largest particle sizes in the baseball rubbing mud mixture. We maintained a gap height of 1 mm for all the experiments, which is an order of magnitude higher than the largest sizes of mud constituents, thereby minimizing the effects of particle confinement on rheology measurements during the shear flow (59).

Samples undergo robust preshear protocol to generate reproducible microstructure prior to probing the material rheology, as shown in our earlier work (38). Steady shear measurements are performed using downward sweeps with shear rates ($\dot{\gamma }$) ranging from 250 to 0.025 s^−1^, where the shear stress is allowed to equilibrate at each $\dot{\gamma }$. Linear viscoelastic moduli are characterized using oscillatory shear experiments performed at the linear rheological regime of the Rubbing Mud sample. After preshear, the oscillatory frequency (*f*) was varied from 15 to 0.015 Hz (angular frequency, *ω*: 100 to 0.01 rad/s), and the elastic moduli ($G^{\prime }$) and viscous moduli ($G^{\prime \prime }$) are measured.

### Soft Tribology.

Soft tribological experiments on Rubbing Mud are performed using custom-designed ball-on-three-plate tribology geometry, attached to the existing rheometer setup (DHR-3, TA Instruments) at ambient temperature and humidity conditions. The bottom steel plates are replaced with custom-made acrylic plates, where the baseball skin slabs (1 cm × 1 cm) (pristine or mudded and dried overnight) are attached to the acrylic using a double-sided tape (Fig. 3B and C, *SI Appendix*, Fig. S1). The top steel ball is replaced with PDMS spheres (radius = 1.27cm) made with Sylgard 184 (Dow Corning) base material. The base to curing agent ratio is maintained at 10:1 (wt/wt%), which gives a PDMS elastic modulus that is comparable to human fingers (45, 46). Finally, the PDMS sphere is dipped in squalene (≥98%, liquid; Sigma-Aldrich), an animal-based oil with similar viscosity as the sebum secreted by human fingers. In addition, squalane has a low vapor pressure, $O(10^{-6})$ Pa (60), which eliminates the possibility of solvent evaporation during high shear experiments (61).

All the soft tribology experiments are performed at constant normal force, $F_{N}=10$ N. Additional data for $F_{N}=1$ N is provided in *SI Appendix*, Fig. S4. Prior to performing tribology experiments, the normal force is set to zero. The PDMS sphere coated with squalene was then lowered manually to make contact with the three acrylic plates with baseball slabs at the desired normal force (*F*_*N*_). All the experiments are performed after the normal forces are stabilized. The samples are subjected to a constant sliding speed *u* and we wait for the torque to equilibrate. The shear force (*F*_*s*_) is estimated from the sliding velocity and the dimensions of geometries involved. The friction coefficient (*μ*) is then estimated as the ratio of shear and normal force as $\mu \equiv F_{s}/F_{N}$. To obtain reliable data for a given sliding speed *u*, both the PDMS sphere and the (three) baseball-on-acrylic plates are swapped out for fresh samples and the experiment is repeated thrice. We perform these experiments for angular speeds varying *u* from 0.01 to 40 rad/s, which translates to a sliding speed of 44 to 179,600 μm/s.

### Nanoscale Adhesion.

The adhesive forces at the microscale were characterized using AFM measurements. An FM Asylum MFP-3D unit (Asylum Research) was used to perform the experiments. A soft cantilever tip (silicon nitride material, NanoAndMore Co. USA) with spring constant of 0.055 N/m was employed in a contact mode at a tapping force of 5 nN. The retraction force of the cantilever is calculated at 100 different locations in a 1 μm × 1μm area to generate the force maps. The areas chosen for this measurement are where sand particles are absent.

### XRD.

XRD analysis was conducted using a Panalytical XPert Powder diffractometer, with data processed through Panalytical HighScore software, using copper (Cu K-*α*) as the X-ray source. The resulting raw data are presented as data points joined by a line, depicting intensity as a function of diffraction angle (*SI Appendix*, Fig. S6).

## Supplementary Material

Appendix 01 (PDF)

## Data Availability

All study data are included in the article and/or *SI Appendix*.
