# Supplementary material for: Soft matter mechanics of baseball’s Rubbing Mud
Source: Proc Natl Acad Sci U S A. 2024 Nov 4;121(47):e2413514121. doi: 10.1073/pnas.2413514121 (PMC11588067; doi:10.1073/pnas.2413514121)
Supplement: Supplementary file 1 — Appendix 01 (PDF) [file pnas.2413514121.sapp.pdf]

Supporting Information for

Soft matter mechanics of baseball's Rubbing Mud

Shravan Pradeep, Xiangyu Chen, Ali Seiphoori, David R. Vann, Paulo E. Arratia, and Douglas J. Jerolmack*

Corresponding Author name.
***E-mail: sediment@sas.upenn.edu**

This PDF file includes:

Figs. S1 to S6

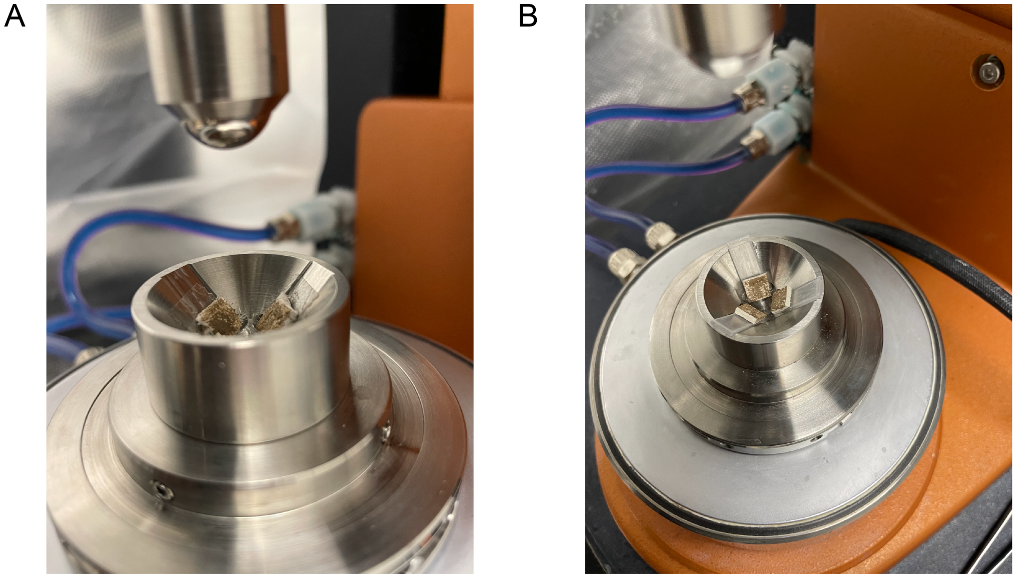

Fig. S1. *(A)* The soft tribology experimental setup with the top geometry (PDMS/steel spheres) attached to the TA Intruments DHR-3 model stress-controlled rheometer. *(B)* The bottom geometry consists of three plates at a vertical angle of 45^0 from the base and a horizontal projected angle of 120^0 between each other. Each acrylic plate is attached to the bottom stainless steel geometry using a double-sided tape. The 2cm x 2cm baseball slabs are attached to the acrylic sheet using a UV glue (3M).

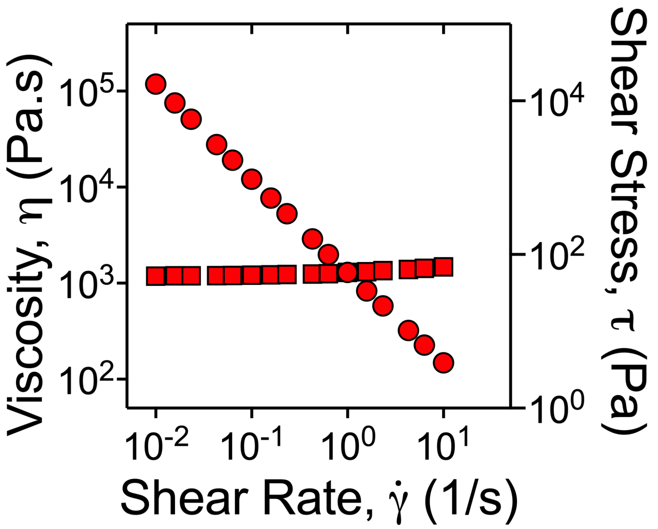

Fig. S2. The steady shear flow curve of pure kaolin suspensions (kaolinite clay particles dispersed in de-ionized water) at $\phi = 0.40$, close to its jamming point of $\phi_J = 0.43$. The plot represents variation in apparent viscosity η and shear stress τ, with change in applied shear rate $\dot{\gamma}$ in a plate-plate rheometry test. This rheological behavior is strickingly similar to the curves of Rubbing Mud.

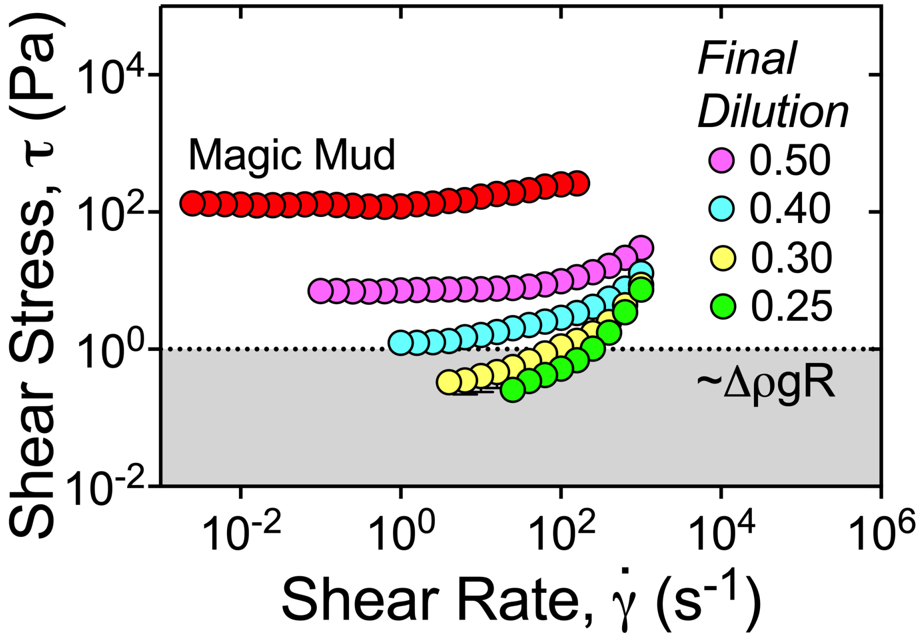

Fig. S3. Steady shear flow curves of Rubbing Mud samples that are diluted with de-ionized water and mixed well. The final mix refers to the dilution ratio where the value = 1.0 for pure Rubbing Mud material. The lower limit of dilution is set by the gravitational stress, $\tau_g \sim \Delta\rho g R$, where R is the largest particle size in the mud. This stress scale sets the lower reliable shear stress below with gravitational stress on higher particles with affect the shear rheology. The divergence of the yield stress shows that the Rubbing Mud is prepared close to its jamming point.

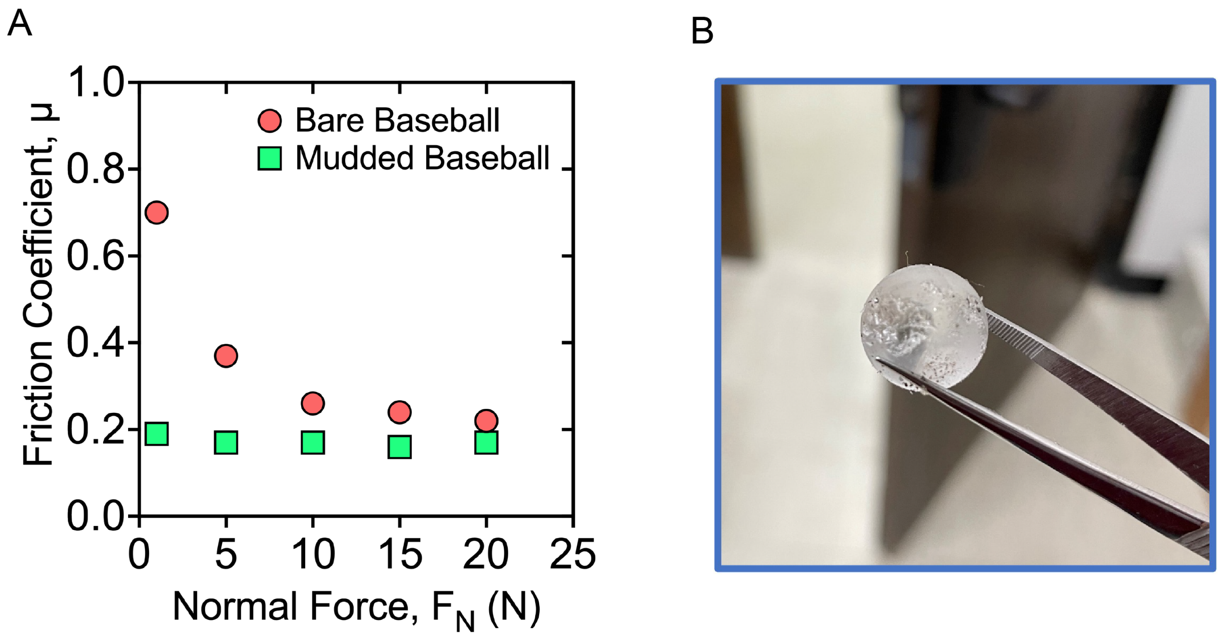

Fig. S4. *(A)* The change in friction coefficient (μ) with respect to change in applied normal force (F_N), with steel ball on baseball plate geometry. The reported values represent the boundary lubrication for dry friction, which remains constant across sliding speeds ranging from 0.05 to 50 rad/s. The μ value for the control bare baseball decreases with increasing F_N. This is speculated to be due to the compression of valley-like features on the surface of the baseball, which impart friction. The μ value plateaus at $F_N \sim 20$ N, where we can assume that the steel ball has compressed baseball surface to the maximum. On the other hand, the mudded surface exhibit no such change in dry boundary friction across the F_N applied. This shows that Rubbing Mud, by covering the pores on the bare baseball mud, reduces the μ, irrespective of the load applied on a steel ball-based experiments. At high normal forces, both bare and mudded baseball show similar friction coefficient ($\mu \approx 0.2$), confirming that the μ from bare baseball comes from these valley-like structures on its surface. *(B)* The wear on the non-lubricated PDMS sphere used under sliding speed of u = 10 rad/s on the mudded baseball surface.

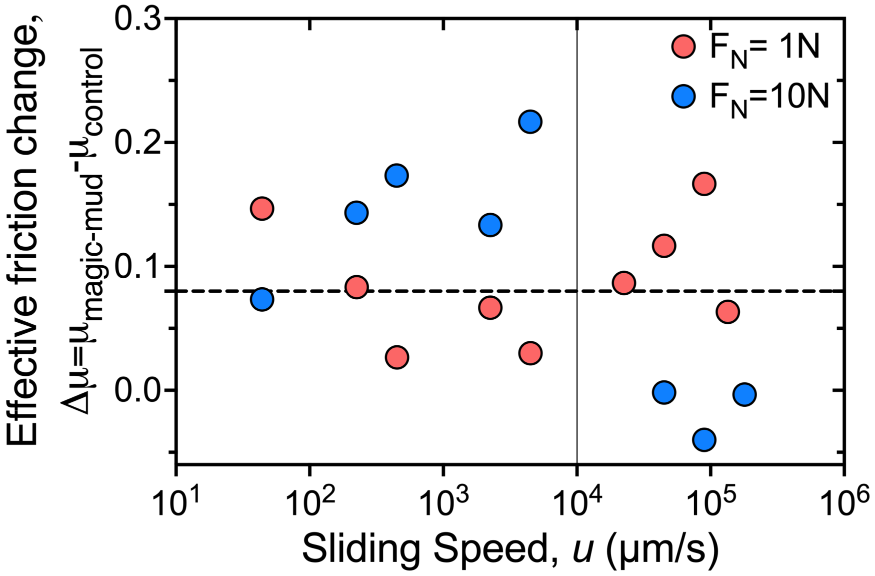

Fig. S5. The change in friction coefficient ($\Delta\mu$), estimated as the change in μ with respect to the control bare baseball, as a function of applied sliding speed u at two normal forces: 1 N and 10 N. At F_N = 10 N, there is a critical speed at $u \approx 10^4$, where the larger particles detaches from the surface lowering the friction similar to bare baseball surface, as shown in the main text. On the other hand, data at F_N = 1 N shows no such trends. The friction values, $\mu = f(u)$, hovers around an average $\Delta\mu \approx 0.08$, indicated by the dashed line. This shows that there is a minimum F_N where the grip from frictional particles is better "felt".

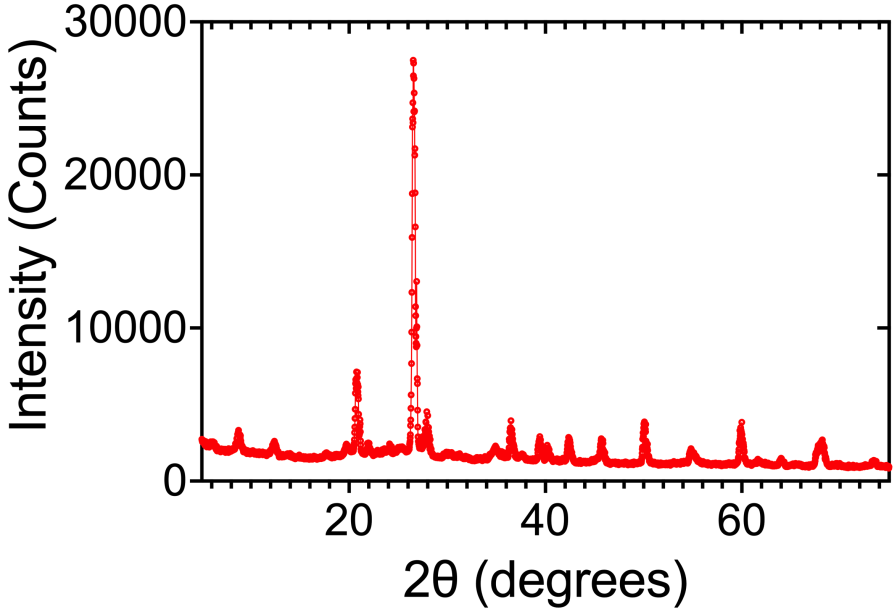

Fig. S6. XRD characterization of Rubbing Mud. The peak at $2\theta \approx 26^0$ corresponds to SiO_2, which corresponds to the large sand and fine silt particles.
